# Application of machine learning approach in emergency department to support clinical decision making for SARS-CoV-2 infected patients

**DOI:** 10.1515/jib-2022-0047

**Published:** 2023-03-07

**Authors:** Nicolò Casano, Silvano Junior Santini, Pierpaolo Vittorini, Gaia Sinatti, Paolo Carducci, Claudio Maria Mastroianni, Maria Rosa Ciardi, Patrizia Pasculli, Emiliano Petrucci, Franco Marinangeli, Clara Balsano

**Affiliations:** School of Emergency Medicine, Interdisciplinary BioMedical group on Artificial Intelligence, IBMAI, Department MeSVA, University of L’Aquila, L’Aquila, Italy; Francesco Balsano Foundation, Via Giovanni Battista Martini 6, 00198, Rome, Italy; Department of Public Health and Infectious Diseases, “Sapienza” University of Rome, Policlinico Umberto I Hospital, Rome, Italy; Department of Anesthesiology, Intensive Care and Pain Treatment, University of L’Aquila, L’Aquila, Italy

**Keywords:** COVID-19, emergency medicine, machine learning, ROX index, SARS-CoV-2

## Abstract

To support physicians in clinical decision process on patients affected by Coronavirus Disease 2019 (COVID-19) in areas with a low vaccination rate, we devised and evaluated the performances of several machine learning (ML) classifiers fed with readily available clinical and laboratory data. Our observational retrospective study collected data from a cohort of 779 COVID-19 patients presenting to three hospitals of the Lazio-Abruzzo area (Italy). Based on a different selection of clinical and respiratory (ROX index and PaO2/FiO2 ratio) variables, we devised an AI-driven tool to predict safe discharge from ED, disease severity and mortality during hospitalization. To predict safe discharge our best classifier is an RF integrated with ROX index that reached AUC of 0.96. To predict disease severity the best classifier was an RF integrated with ROX index that reached an AUC of 0.91. For mortality prediction the best classifier was an RF integrated with ROX index, that reached an AUC of 0.91. The results obtained thanks to our algorithms are consistent with the scientific literature an accomplish significant performances to forecast safe discharge from ED and severe clinical course of COVID-19.

## 1 Introduction

The pandemic caused by severe respiratory acute syndrome coronavirus 2 (SARS-CoV-2) was declared global emergency on 11th March 2020, by the World Health Organization (WHO) [1]. Since then, the development and distribution of Coronavirus Disease 2019 (COVID-19) mRNA vaccines, and the efforts made to select more effective antiviral and immunomodulatory therapies have significantly reduced the incidence of severe disease and mortality among patients [2–4].

Although these progresses have changed the face of pandemic, some concerns remain about threats related to SARS-CoV-2 [5].

By example, some countries have not adopted a common global response to SARS-CoV-2 infection and vaccination inequities are manifest [6]. As of July 2022, the 85% of vaccine doses have been administrated in high income countries; in low-income countries the accessibility to vaccination is the principal cause of unvaccinated patients, especially in the African continent, where for some countries the fully vaccinated protocol was administrated to the 10–20% of population [7–10]. This scenario confirms that recurrence of viral outbreak remains a concrete challenge, especially in countries where the doses of vaccines administered are inadequate.

An early clinical prediction of clinical outcome could be beneficial for the patient’s management and for the optimal allocation of healthcare resources [11, 12]. As we previously experienced, the clinical burden was higher in non-metropolitan areas than metropolitan ones, where the hospitals and medical services were less prepared for a viral outbreak [13]. Artificial intelligence and related tools could overcome these issues by supporting physicians in clinical routine. Accordingly, several clinical indexes, machine learning (ML) models and apps have been demonstrated to be a powerful approach for predicting hospitalization, disease severity and mortality in ED [14–18].

We performed an overview of several state-of-the-art ML classifiers that, starting from routine clinical and laboratory analyses, could support physician’s decision making in achieving a safe discharge, as well as in forecasting disease severity and mortality risk of SARS-CoV-2 infected patients. All the ML classifiers, used in the study, were compared with the indexes to assess the risk of severe disease or death, commonly used in ED.

The work presented in this paper is part of a larger research project whose aim was to design, develop and test a decision support system, called C19DSS (COVID-19 Decision Support System), based on ML predictors, that should assist physicians in clinical decision making [19].

## 2 Materials and methods

### 2.1 Study design and sample

This study retrospectively analyzed a cohort of SARS-CoV-2 infected patients presenting to ED, to collect a dataset for devising the ML models. Biochemical and clinical data of 779 adults (age > 18 years), affected by SARS-CoV-2, admitted during the first two waves to three hospitals of Lazio-Abruzzo area, were collected and analyzed. Missing data were managed by automatic imputation as described in the section below.

Demographic, clinical, and biochemical variables were collected by a physician, during the first presentation at each ED.

The clinical outcomes were safe discharge, disease severity at seventh day of hospitalization and mortality during hospitalization. Data were collected at three different times: at ED presentation (T0), at the seventh day of hospitalization (T7) and at hospital discharge (TD). The disease severity was interpreted by following the interim guidance released by the WHO [20]. WHO ordered the severity of the infectious disease in “mild” (WHO score 0), “moderate” for patients who no required supplemental oxygen (WHO score 1), and “severe” for all patients who needed non-invasive ventilation (NIV) or high-flow oxygen requirement or continuous positive airway pressure (CPAP) (WHO score 2), and “critical” for patients who needed invasive ventilation or multi-organ support (WHO score ≥3). The safe discharge was a composite endpoint considering for all enrolled patients: PaO_2_/FiO_2_ ratio > 274, PaO_2_ > 65 mmHg, no oxygen supply during hospitalization, length of hospitalization < 10 days and no adverse events during hospitalization (endotracheal intubation, disease progression to severe or critical disease, admission to high dependency unit, intensive care unit, mortality). The cut-off of PaO_2_/FiO_2_ ratio > 274 was chosen considering the results reported in our previous manuscript [21]. The safe discharge outcome reported in the dataset was then revised, so to represent the correct decision that should have been taken at admission time. The mortality risk and the severity disease risk were retrospectively evaluated, applying respectively the 4C Mortality Score for COVID-19 (4C MS) and the quick COVID-19 Severity Index (qCSI) [22, 23].

The study was approved by the Internal Review Board of our Institution (IRB 06/2021, protocol number 28,958) and registered on clinicaltrial.gov, protocol number NCT04825301. The study was conformed to the Transparent Reporting of a multivariable prediction model for Individual Prognosis or Diagnosis (TRIPOD) statement and should be considered a prediction model study type 2a [24].

### 2.2 Missing data imputation

To devise the models in case of missing data, the dataset was pre-processed through missing data imputation system [25, 26]. In detail, predictive mean matching for numeric data and logistic regression imputation for categorical data (with 2 levels) was used [27].

### 2.3 Predictors

To devise the ML models, both continuous and categorical variables were used. Heart rate (HR), respiratory rate-oxygenation (ROX) index, alanine aminotransferase (ALT), aspartate aminotransferase (AST), creatinine, C-reactive protein (CRP), lactic dehydrogenase (LDH), platelet count (PLT), white blood cells count (WBC), neutrophil count (NEU), lymphocyte count (LIN), eosinophil count (EOS), basophil count (BAS), neutrophil lymphocyte ratio (NLR), platelet lymphocyte ratio (PLR), international normalized ratio (INR), D-dimer (DD), standard blood gas analysis variables and PaO_2_/FiO_2_ ratio have been included as continuous variables. Sex at birth, fever (or not) and cough (or not) have been considered as categorical variables.

### 2.4 Outcomes

Primary outcome was the prediction (i.e., safe/risk) of safe discharge; secondary outcomes were the prediction of disease severity at seventh day (i.e., severe/mild) and mortality during hospitalization (i.e., present/absent).

### 2.5 Statistical analysis and ML models

The descriptive analyses consist of frequency tables, medians and IQRs. Data reported in Supplementary Table 1 have been analyzed using one way ANOVA with post-hoc Fisher LSD tests for multiple comparison.

Based on a different selection of predictors, several models with complete set of predictors and with set of predictors were devised, details are reported in Supplementary Table 3.

The ML task for all outcomes is binary classification. The following different models were tried from the available data: decision tree (DT), random forest (RF) and gradient boosting machines (GBM) [28–30]. For all models and outcomes, the dataset was split into train and test (with 75% of data going for training, 25% for testing), used 10-fold cross-validation, tuned each classifier according to its specific hyperparameters, calculated the confusion matrix (with the most useful associated statistics, i.e., accuracy, Cohen’s K, sensitivity, specificity) and the ROC curve (with the related AUC) [31]. To consider data imbalance, we also adopted RUSBoost as hybrid sampling/boosting algorithm [32].

In addition, to improve the explainability of the models, the variables importance was calculated and presented as graphs [33].

To compare the ML models (for the disease severity and mortality risk) with the standard clinical practice, the qCSI and 4C MS indexes was dichotomized. The confusion matrices (with the same associated statistics of the ML models) were calculated and finally, the results were compared with the predictions of the corresponding ML models, i.e., severity and mortality, respectively.

All statistical analyses and ML models discussed in the paper were calculated with RStudio 2022.07.2–576 running R4.2.1 for Linux, using the “caret” and “mice” packages [27, 34]. Due to the restrictions decided by the Internal Review Board, we are unable to publicly share the study data.

## 3 Results

### 3.1 Sample description

The final dataset included 779 patients; demographic and clinical characteristics and principal biochemical markers for the study population are reported in Supplementary Table 1. Data record was not complete for 283 patients, whereas 496 patients have got a complete data record. The study population presented a median age of 65 years (IQR 55–75), 41.3% were female, more than three comorbidities were present in 19% of the cases. The median value of CRP was 4.84 mg/dL (IQR 1.58–10.89), of LDH was 302 IU/L (IQR 227–389.5), of platelet was 202 10^3^/mcl (IQR 160–265), of WBC was 6.27 10^9^/L (IQR 4.60–9.24), of neutrophil count was normal 4.55 10^9^/L/L (IQR 3.11–7.60), of lymphocytes count was 0.92 10^9^/L/L (IQR 0.64–1.33). D-Dimer was tested in D-Dimer Unit (DDU), its median value was 0.81 mcg/mL DDU (IQR 0.47–1.50). Respiratory parameter as PaO_2_ had a median value of 70.2 mmHg (IQR 59–83), the PaO_2_/FiO_2_ ratio 308 (IQR 223–365) and the ROX index 20.4 (IQR 13–20.9). Patients with severe disease or deceased were elder and characterized by higher values of CRP, LDH, D-Dimer, and progressive lower values for the PaO_2_/FiO_2_ ratio and ROX index, compared to the safe discharge population.

At presentation in ED, 72.6% of patients reported fever and 54.1% referred cough; the clinical spectrum of disease of the whole cohort is outlined in Supplementary Table 2. At presentation in ED, SARS-CoV-2 patients presented mild (26.3%), moderate (38.7%), severe (28.6%), critical (6.4%) symptoms. In Supplementary Table 2 are reported the distribution of safe discharge, severe disease and died population considering the WHO interim guidance (see material and methods).

### 3.2 Machine learning models

As previously described, based on a different selection of predictors we devised several models, the results of all the prediction models developed are reported in Supplementary in Table 4.

**Figure 1:**
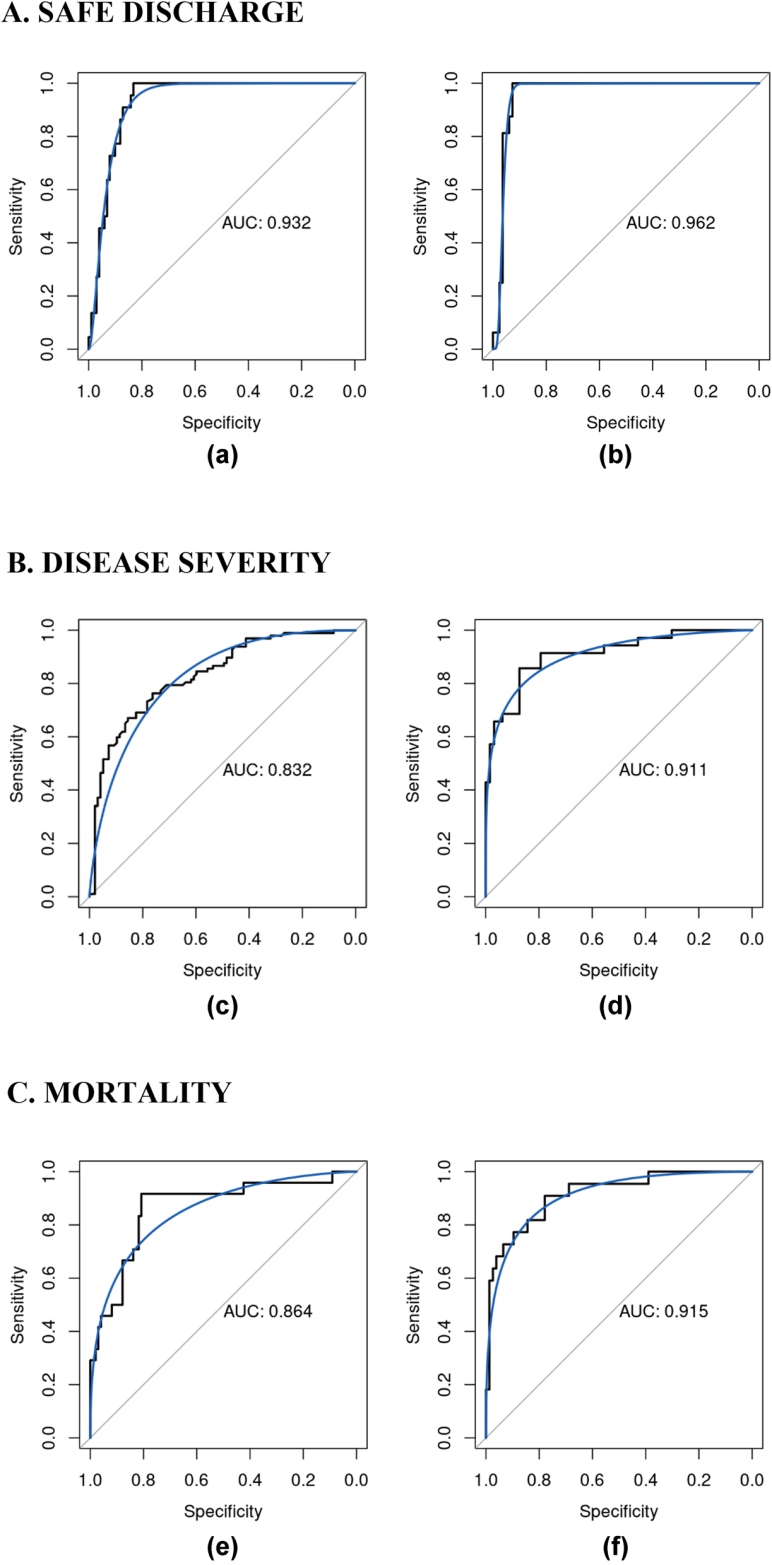
ROC curves of the best models (in terms of AUC) for (A) safe discharge: (a) PaO_2_/FiO_2_ ratio RUSBoost model – RF, (b) ROX index RUSBoost model – RF; (B) disease severity: (c) PaO_2_/FiO_2_ ratio model – RF, (d) ROX index RUSBoost model – RF; (C) mortality: (e) PaO_2_/FiO_2_ ratio RUSBoost model – GBM, (f) ROX index RUSBoost model – RF.

**Figure 2:**
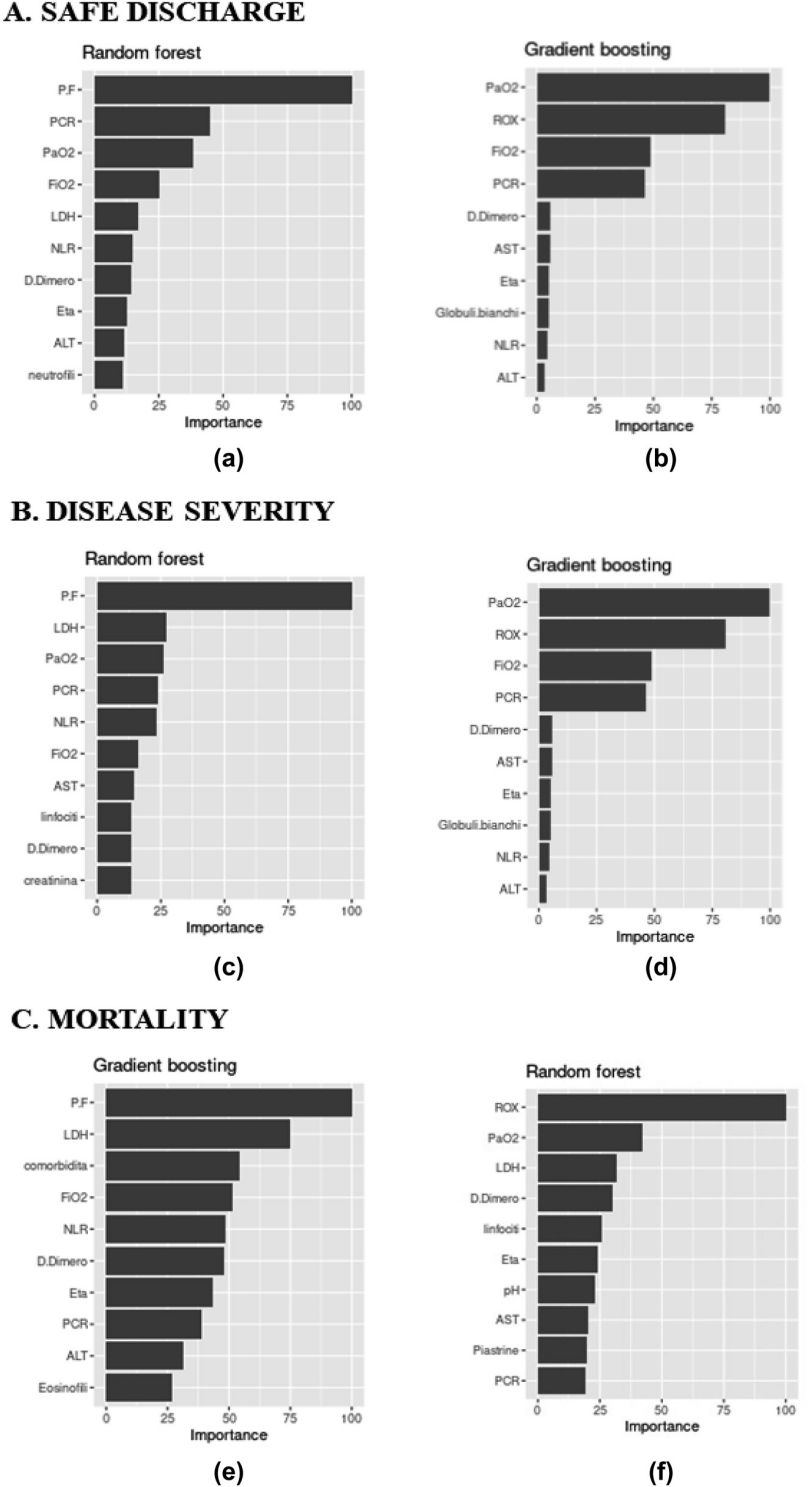
Variables ranked by importance for the different outcomes for (A) safe discharge: (a) PaO_2_/FiO_2_ ratio model – RF, (b) ROX index model – GBM; (B) disease severity: (c) PaO_2_/FiO_2_ ratio model – RF, (d) ROX index model – RF; (C) mortality: (e) PaO_2_/FiO_2_ ratio model – GBM, (f) ROX index model – RF.

Tables 1a and 1b report the main statistics as coming from the confusion matrices for the best classifiers selected: the ROX index models and the PaO_2_/FiO_2_ ratio models. Figure 1 instead contains all ROC curves with the related AUCs, for the best classifiers (in terms of AUC), for the different outcomes. Finally, Figure 2 reports the importance of the first ten variables, ranked by importance, for the different outcomes, for the best classifiers (in terms of AUC).

**Table 1a:** PaO_2_/FiO_2_ ratio models: main statistics for all outcomes and classifiers.

	PaO_2_/FiO_2_ ratio models				PaO_2_/FiO_2_ ratio, RUSBoost models			
	Acc.	Sens.	Spec.	AUC	Acc.	Sens.	Spec.	AUC
**Safe discharge**								
DT	0.824	0.842	0.770	0.858	0.837	0.811	0.954	0.927
RF	**0.829**	0.869	**0.780**	**0.894**	0.837	0.802	**1.00**	**0.932**
GBM	0.824	**0.876**	0.666	0.882	**0.845**	**0.831**	0.909	0.908
**Disease severity**								
DT	0.742	0.732	**0.752**	0.766	0.699	**0.729**	0.653	0.773
RF	0.757	0.783	0.732	**0.832**	**0.739**	**0.729**	**0.755**	**0.815**
GBM	**0.762**	**0.804**	0.721	0.827	0.707	0.689	0.734	0.773
**Mortality**								
DT	0.840	0.944	0.290	0.689	0.772	0.818	0.583	0.759
RF	**0.876**	**0.969**	0.387	0.842	0.788	0.808	**0.708**	0.856
GBM	0.860	0.944	**0.419**	**0.844**	**0.804**	**0.838**	0.666	**0.864**

The best result is reported as bold value.

**Table 1b:** ROX index models: main statistics for all outcomes and classifiers.

	ROX index models				ROX index, RUSboost models			
	Acc.	Sens.	Spec.	AUC	Acc.	Sens.	Spec.	AUC
**Safe discharge**								
DT	**0.928**	0.939	**0.875**	0.778	**0.938**	**0.939**	0.937	0.960
RF	**0.928**	**0.963**	0.750	0.879	0.928	0.914	**1.00**	**0.962**
GBM	0.898	0.951	0.625	**0.898**	0.898	0.890	0.937	0.955
**Disease severity**								
DT	0.826	**0.857**	0.809	0.709	0.816	0.809	0.828	0.877
RF	0.816	0.742	**0.857**	**0.806**	**0.846**	**0.841**	0.857	**0.911**
GBM	**0.836**	0.800	**0.857**	0.800	0.795	0.730	**0.914**	0.910
**Mortality**								
DT	0.827	**0.974**	0.318	0.735	0.787	0.779	**0.818**	0.864
RF	**0.828**	**0.974**	0.318	**0.860**	**0.858**	**0.883**	0.772	**0.915**
GBM	**0.828**	0.935	**0.454**	0.844	0.777	0.792	0.727	0.878

**PaO_2_/FiO_2_ ratio models.** For safe discharge, RF is the most accurate in almost all the statistics, except for sensitivity, where GBM performs slightly better. The results for disease severity showed that RF performed with the best AUC; conversely, GBM has slightly superior sensitivity and accuracy. Regarding mortality during hospitalization, GBM reached the best AUC and specificity, on the other hand, RF performed the best accuracy and sensitivity (Table 1a and Figure 1).

**ROX index models.** For safe discharge, GBM has the best AUC. On the other hand, RF demonstrated the highest sensitivity and accuracy. The results for disease severity showed that RF is the most accurate model, with the highest specificity and the best AUC. Conversely, GBM has the best accuracy. In connection with mortality during hospitalization, RF is the best classifier in terms of all statistics, except for specificity, where GBM performs slightly better (Table 1b and Figure 1).

### 3.3 Comparison of ML models with standard clinical practice

**Figure 3:**
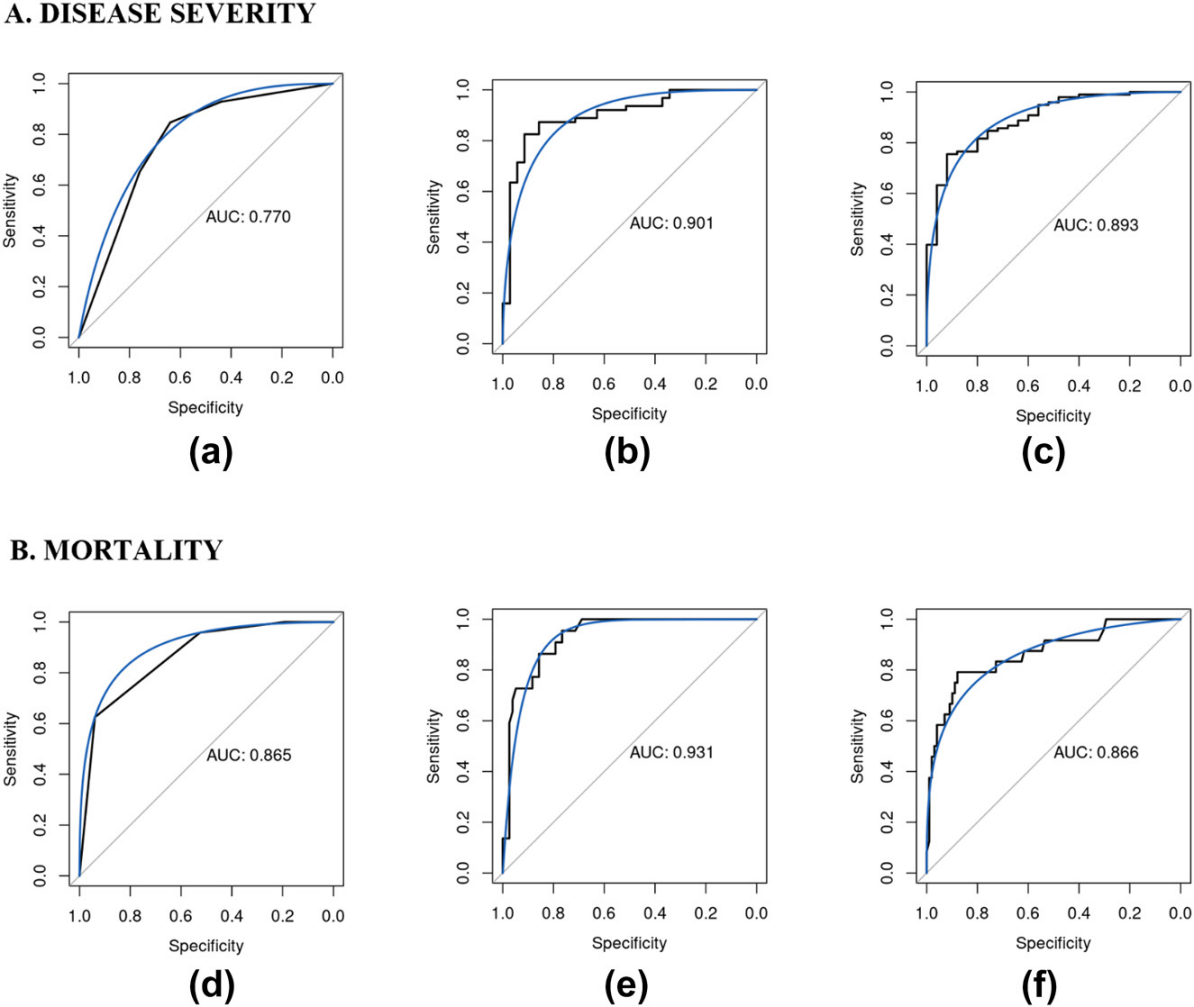
ROC curves and AUCs for (A) disease severity: (a) quick Covid Severity Index (qCSI), (b) ROX index model – GBM, (c) PaO_2_/FiO_2_ ratio model – GBM; (B) Mortality: (d) 4C Mortality Score (4C MS), (e) ROX index model – RT, (f) PaO_2_/FiO_2_ ratio model – RF.

Table 2 contains the main statistics of the best classifiers and the indexes commonly used in clinical practice, for both disease severity and mortality. Figure 3 depicts the comparison in terms of ROC curves and AUCs. As mentioned earlier, given that the indexes could be computed only for the complete cases, the comparison is reported with the models devised from the same subset of observations. As previously mentioned, the main statistics for complete cases are reported in Supplementary Table 4. The results show that our models for predicting mortality outperforms the 4C MS in all statistics. On the other hand, with respect to qCSI, our models for predicting disease severity showed higher accuracy, specificity and AUC, but a smaller sensitivity.

**Table 2:** Main statistics of the standard indexes for disease severity and mortality, regarding our best classifiers.

	Acc.	Sens.	Spec.	AUC
**Disease severity**				
PaO_2_/FiO_2_ ratio model – GBM	**0.846**	0.600	**0.908**	0.893
ROX index model – GBM	0.836	0.800	0.857	**0.917**
qCSI	0.805	**0.958**	0.535	0.770
**Mortality**				
PaO_2_/FiO_2_ ratio model - RF	**0.878**	0.960	**0.542**	0.866
ROX index model – RF	0.828	**0.974**	0.318	**0.906**
4C MS	0.618	0.958	0.535	0.865

### 3.4 External validation

The best prediction models were selected and integrated into a system; further details are described in the paragraph below (see **COVID-19 Decision Support System**). During the fourth wave, the system was used by the physicians of different institutions and was applied to patients with infection by SARS-CoV-2. So far, the validation cohort is made up of 57 patients. The mortality risk prediction model showed an accuracy of 0.94 for both GBM and RF models, whereas the models about safe discharge returned an accuracy of 0.70 and 0.60 (RF and GBM respectively).

Therefore, as expected, the system confirms its high sensitivity, but a lower overall accuracy [34, 35].

### 3.5 COVID-19 Decision Support System (C19DSS)

We developed the COVID-19 Decision Support System (C19DSS) to enable physicians to effectively use the previous models. The system is made up of a smartphone app used by clinicians, and a server that provides the “intelligence” to the app.

In short, the app is made up of four activities (see Supplementary Figure 1). The first activity is the dashboard, that summarizes the database content and the server connection status. The second activity contains the patient list, plus a set of filters and a button to add a new patient. The third screen shows how to enter the laboratory/clinical data of a new patient, and the button to request the classification to the server. The fourth screen depicts how to edit the patient data, as well as request the classification to the server or delete the patient from the local database.

When the classification process is activated, the app opens an encrypted connection to the server, sends the laboratory data and the ID of the patient (so, no personal data is communicated over the network) to the classification service. Then, the server applies the correct model, depending on the request and on the received data, stores the received data for further analyses, and returns the classification results to the app. The activities briefly described above were designed, developed, tested and redesigned following the UCD methodology, i.e., physicians were involved from the very beginning phases of the design, and then we adapted and improved the design/implementation according to consecutive cycles of usability tests. Physicians reported that the application was easy ed intuitive to use; the process of data entry and classification did not hamper the normal ED work routine. More details on the system, its architecture and on the usability results can be found in a further papers of our research group [19].

## 4 Discussion

This study was finalized to devise ML methods, fed by routine clinical and laboratory analyses, to be used by physicians to manage safe discharge or hospitalization of unvaccinated COVID-19 patients. In particular, the models aim at predicting three different clinical outcomes: safe discharge, severe disease, at the seventh day after medical presentation, and mortality during hospitalization.

To compare our results with the literature, we proceeded as follows. First, we selected the highest accurate models, coming from peer-reviewed papers, that make use only of clinical data for the classification of the disease without the support of CT or X-ray images [36–39]. The best model found in scientific literature for hospital admission was an RF model that reached an AUC = 0.82. In our research, the ROX index and PaO_2_/FiO_2_ ratio models used for the prediction of safe discharge reached an AUC of 0.962 and 0.932, respectively. For severity prediction a CatBoost was the best model and reached an AUC equal to 0.84, versus the 0.911 of ROX index model and 0.832 of PaO_2_/FiO_2_ ratio model. For mortality prediction, the best classifier was a Support Vector Machines (SVM) model that reached an AUC of 0.91, versus 0.864 of ROX index (RF) model and 0.915 of PaO_2_/FiO_2_ ratio (GBM) model [38, 40, 41]. As can be seen, the RUSboost approach used to consider data imbalance returned the best results, with the only exception of predicting disease severity, in the case of PaO_2_/FiO_2_ ratio, where a standard RF model achieved better performances.

To our knowledge this is the first study that integrated the ROX index into a machine learning model for clinical prediction [42, 43]. Compared to our PaO_2_/FiO_2_ ratio model the ROX index model was equal and even slightly superior in all the statistics, and it reached the best performance in the safe discharge model (Figure 1). Although the PaO_2_/FiO_2_ ratio is the most validated and widely used index for respiratory decompensation, the ROX index is simple to use because it needs only readily available clinical data (RR, SpO_2_ and FiO_2_) to be calculated [44, 45]. For these properties this simpler model can better help to quickly prioritize patients in the pre-hospital scene and in all the area with limited health resources. Finally, with respect to the standard clinical practice, our models outperform the 4C MS and qCSI indexes [22, 23].

With respect to other models available in the literature, ours are tailored to the clinical and laboratory data that are routinely collected at the first medical presentation in ED.

As expected, the performance in the external validation demonstrated a lower accuracy for both mortality and safe discharge prediction. The reason can be twofold. First, the external validation was conducted on a smaller dataset. Second, the patients of the external validation were enrolled on a second wave of SARS-CoV-2 pandemic respect to those of the internal validation. Accordingly, the evolution and changes of dominant SARS-CoV-2 variants, between the different waves, could have taken a place in this reduced accuracy [46].

## 5 C19DSS future development

The C19DSS quickly predicted the prognosis in unvaccinated patients with pneumonia due to SARS-CoV-2 infection and, to the best of our knowledge, is the first attempt to provide an accurate prediction of safe discharge from ED. As future work, given the rapid dynamic of the pandemic, our AI-driven prediction models need to be updated to maintain high performance. To this aim, we have programmed to update the models, so that they will take into account the patient’s immunization status against SARS-Cov-2 infection, so to improve prediction also in vaccinated, partially vaccinated and immunocompromised patients.

## 6 Limitations

Besides the limited size of the dataset and the constraint of using only routine clinical and laboratory data to devise the models, the performances of our models are in line and even better than the best prediction models in scientific literature. Furthermore, the prediction models are devised from a cohort of unvaccinated patients, and therefore its applicability is limited to countries with limited access to vaccines. We look forward to subsequent large sample studies and external validations in undeveloped countries.

## 7 Conclusions

We proposed a novel AI-driven prediction model integrated with the ROX index to forecast safe discharge and mortality in unvaccinated COVID-19 patients.

We conceive that, in the next future, integration of prediction models in emergency care routine could improve healthcare assistance and represent an opportunity to make the people safer.

## Supplementary Material

Supplementary Material Details
