# Supplementary material for: Application of machine learning approach in emergency department to support clinical decision making for SARS-CoV-2 infected patients
Source: J Integr Bioinform. 2023 Mar 7;20(2):20220047. doi: 10.1515/jib-2022-0047 (PMC10561065; doi:10.1515/jib-2022-0047)
Supplement: Supplementary file 1 — Supplementary Material Details [file j_jib-2022-0047_suppl_001.docx]

**Application of machine learning approach in Emergency Department to support clinical decision making for SARS-CoV-2 infected patients**

Nicolò Casano^1^; Silvano Junior Santini^1,2^; Pierpaolo Vittorini^1,2^; Alessandro Grimaldi^3^; Paolo Carducci^4^; Claudio Maria Mastroianni^5^; Maria Rosa Ciardi^5^; Patrizia Pasculli^5^; Emiliano Petrucci^6^; Franco Marinangeli^6^; Clara Balsano^1,2*^.

1. School of Emergency Medicine, Interdisciplinary BioMedical group on Artificial Intelligence, IBMAI, Department MeSVA, University of L’Aquila, L'Aquila, Italy

2. Francesco Balsano Foundation, Via Giovanni Battista Martini 6, 00198, Rome, Italy

3. Department of Infectious Disease, San Salvatore Hospital, L'Aquila, Italy

4. Department of Pneumology, San Salvatore Hospital, L'Aquila, Italy

5. Department of Public Health and Infectious Diseases, "Sapienza" University of Rome, Policlinico Umberto I Hospital, Rome, Italy

6. Department of Anesthesiology, Intensive Care and Pain Treatment, University of L'Aquila, L’Aquila, Italy

***Correspondence**: Prof. Clara Balsano University of L’Aquila, Piazza S. Salvatore Tommasi 1, 67100, Coppito, L’Aquila, Italy clara.balsano@univaq.it. telephone number +39 0862434774

| **Continuous variables** | | | | |  |
| --- | --- | --- | --- | --- | --- |
| **Parameter** | **Study population**  **(n=779)** | **Severe and critical disease population**  **(n=295)** | **Dead population**  **(n=126)** | **Safe discharge population**  **(n=195**) |  |
| Age (years) | 65 (55 - 75) | 67 (59 - 76) | 72.50 (67 - 82.75)* | 58 (48 - 70)***^;###^ |  |
| HR (bpm) | 85 (76 - 96) | 85 (78 - 95) | 88 (80 - 100) | 84 (73 - 98) |  |
| FR (apm) | 18 (16 - 22) | 20 (17 - 24) | 20 (18 - 25) | 16 (14 - 18)***^;###^ |  |
| ROX index | 20.4 (13 - 20.9) | 16 (9 - 23) | 10.5 (7.2 - 17.7)*** | 29 (26.9 - 33.3)***^;###^ |  |
| SpO_2_ (%) | 93 (90 - 96) | 85 (78 - 95) | 89 (84 - 94)** | 96 (95 - 97)***^;###^ |  |
| FiO_2_ | 0.21 (0.21 - 0.28) | 0.21 (0.21 - 0.5) | 0.40 (0.21 - 0.60) | 0.21 (0.21 - 0.21) |  |
| ALT (IU/mL) | 25 (17 - 40) | 29 (19 - 46) | 28 (17 - 50) | 21 (14 - 33) |  |
| AST (IU/mL) | 29 (21 - 40) | 33 (25 - 46) | 36 (24 - 57) | 23 (18 - 32) |  |
| Creatinine (mg/dL) | 0.89 (0.74 - 1.09) | 0.93 (0.78 - 1.18) | 1.04 (0.83 - 1.43) | 0.8 (0.7 -1.0) |  |
| CRP (mg/dL) | 4.84 (1.58 - 10.89) | 8.96 (4.51 - 14.08) | 10.29 (6.34 - 16.84) | 1.61 (0.51 - 3.87)***^;###^ |  |
| LDH (IU/L) | 302 (227 - 389.5) | 357 (286 - 481) | 430 (310 - 591)** | 236 (193 - 307)***^;###^ |  |
| PLT (103/mcl) | 202 (160 - 265) | 205 (159 - 267) | 196 (136 - 283) | 203 (161 - 259) |  |
| WBC (109/L) | 6.27 (4.60 - 9.24) | 7.40 (5.31 - 10.57) | 8.37 (5.77 - 11.32) | 5.5 (4.21 - 7.23) |  |
| NEU# (109/L) | 4.55 (3.11 - 7.60) | 5.90 (3.65 - 8.61) | 7.17 (4.22 - 9.64) | 3.63 (2.66 - 4.92) |  |
| LIN# (109/L) | 0.92 (0.64 - 1.33) | 0.79 (0.58 - 1.06) | 0.71 (0.49 – 0.99) | 1.17 (0.81 - 1.60) |  |
| MON# (109/L) | 0.38 (0.26 - 0.54) | 0.38 (0.26 - 0.55) | 0.36 (0.25 - 0.49) | 0.36 (0.26 - 0.49) |  |
| EOS# (109/L) | 0.01 (0 - 0.03) | 0 (0 - 0.01) | 0 (0 - 0.01) | 0.02 (0.01 - 0.05) |  |
| BAS# (109/L) | 0.01 (0.01 - 0.03) | 0.01 (0.01 - 0.02) | 0.01 (0.01 - 0.03) | 0.02 (0.01 - 0.03) |  |
| NLR | 4.93 (2.79 - 9.31) | 7.38 (4.12 - 12.10) | 9.10 (5.26 - 15.22) | 3.25 (2.03 - 4.94) |  |
| PLR | 215 (149 - 334) | 248 (170 - 404) | 293 (161 - 446) | 187 (138 - 280) |  |
| INR | 1.05 (1.0 - 1.13) | 1.09 (1.01 - 1.17) | 1.12 (1.04 - 1.23) | 1.03 (0.98 - 1.08) |  |
| D-Dimer (mcg/mL DDU) | 0.81 (0.47 - 1.50) | 0.98 (0.61 - 1.83) | 1.50 (0.98 - 2.46)* | 0.58 (0.37 - 0.99)***^;###^ |  |
| pH | 7.46 (7.43 - 7.49) | 7.47 (7.43 - 7.5) | 7.45 (7.40 - 7.49) | 7.46 (7.43 - 7.49) |  |
| PaO_2_ (mmHg) | 70.2 (59 - 83) | 60.7 (51.8 - 73) | 53.9 (48.2 - 71.7) | 82 (73 - 92) |  |
| PaO_2_/FiO_2_ ratio | 308 (223 - 365) | 233 (146 - 293) | 163 (108 - 255) *** | 390 (347 - 438) ***^;###^ |  |
| **Categorical variables** |  |  |  |  |  |
| **Characteristic** | **Study Population**  **(n=779)** | **Severe and critical disease population**  **(n=295)** | **Dead**  **population**  **(n=126)** | **Safe discharge population**  **(n=195**) |  |
| Female | 321 (41.3%) | 102 (34.5%) | 41 (32.5%) | 93 (47%) |  |
| Death | 126 (16%) | 93 (31.5 %) | 126 (100%) | - |  |
| Fever | 565(72.6%) | 215 (72.8 %) | 92 (73%) | 150 (76%) |  |
| Cough | 422 (54.1%) | 200 (67.7%) | 82 (65%) | 101 (52%) |  |
| No comorbidity | 239 (30.0%) | 64 (21.6 %) | 9 (7%) | 94 (48%) |  |
| 1 Comorbidity | 242 (31.7%) | 91 (30.8 %) | 35 (27%) | 58 (29%) |  |
| 2 Comorbidities | 150 (19.3%) | 63 (21.3 %) | 33 (26%) | 24 (13%) |  |
| ≥3 Comorbidities | 148 (19%) | 77 (26%) | 49 (40%) | 19 (10%) |  |

**Table 1.** Categorical (count and percentage) and continuous variables (median, 1^st^ and 3^rd^ quartile). In case of a statistically significant comparison between safe discharge, severe/critical disease, and dead samples, we use: * vs Severe and critical disease population, # vs Dead population; * → *P* < .05; ** → *P*<.01; *** → *P* < .001; ### → *P* <.001.

|  |  | **WHO** | | | |
| --- | --- | --- | --- | --- | --- |
|  | **Time** | **0**  **(mild)** | **1**  **(moderate)** | **2**  **(severe)** | **≥3**  **(critical)** |
| **Study population**  **(n=779)** | T0 | 207 (26.3%) | 300 (38.7%) | 223 (28.6%) | 49 (6.4%) |
|  | T7 | 223 (28.6%) | 261 (31.4%) | 265 (25%) | 102 (13.0%) |
| **Safe discharge population**  **(n=195)** | T0 | 113 (57.5%) | 82 (42.5%) | 0 (0%) | 0 (0%) |
|  | T7 | 130 (66.7%) | 65 (33.3%) | 0 (0%) | 0 (0%) |
| **Severe and critical disease population**  **(n=295)** | T0 | 7 (2.3 %) | 78 (26.7 %) | 162 (56%) | 45 (15%) |
|  | T7 | 0 (0%) | 0 (0%) | 193 (65.5%) | 102 (34.5 %) |
| **Died population**  **(n=126)** | T0 | 5 (3.9%) | 27 (21.4%) | 62 (49.2%) | 32 (25.5%) |
|  | T7 | 6 (4.7%) | 26 (20.6%) | 23 (18.2%) | 71 (56.5%) |

**Table 2.** WHO classification at ED presentation (T0) and after 7^th^ day of hospitalization (T7).

|  | | | | |
| --- | --- | --- | --- | --- |
| **Parameter** | ***PaO_2_/FiO_2_ ratio model*** | ***ROX index model*** | ***ROX index routine model*** | ***ROX index pre-hospital model*** |
| Age (years) | **X** | **X** | **X** | **X** |
| HR (bpm) | **X** | **X** | **X** | **X** |
| FR (apm) |  | **X** | **X** | **X** |
| Comorbidities (n°) | **X** | **X** | **X** | **X** |
| FiO_2_ | **X** | **X** | **X** | **X** |
| ALT (IU/mL) | **X** | **X** |  |  |
| AST (IU/mL) | **X** | **X** |  |  |
| Creatinine (mg/dL) | **X** | **X** |  |  |
| CRP (mg/dL) | **X** | **X** |  |  |
| LDH (IU/L) | **X** | **X** | **X** |  |
| PLT (103/mcl) | **X** | **X** | **X** |  |
| WBC (109/L) | **X** | **X** | **X** |  |
| NEU# (109/L) | **X** | **X** | **X** |  |
| LIN# (109/L) | **X** | **X** | **X** |  |
| MON# (109/L) | **X** | **X** | **X** |  |
| EOS# (109/L) | **X** | **X** | **X** |  |
| BAS# (109/L) | **X** | **X** | **X** |  |
| NLR | **X** | **X** | **X** |  |
| PLR | **X** | **X** | **X** |  |
| INR | **X** | **X** | **X** |  |
| D-Dimer (mcg/mL DDU) | **X** | **X** | **X** |  |
| pH | **X** | **X** |  |  |
| ROX index |  | **X** | **X** | **X** |
| SpO_2_ (%) |  | **X** | **X** | **X** |
| PaO_2_ (mmHg) | **X** | **X** |  |  |
| PaO_2_/FiO_2_ ratio | **X** |  |  |  |

**Table 3.** Variables used for the prediction by different models, where the **X** is present the variable is included.

|  | **Complete cases** | | | | **Missing data imputation** | | | |  |  |  |  |  |  |  |  |  |  |
| --- | --- | --- | --- | --- | --- | --- | --- | --- | --- | --- | --- | --- | --- | --- | --- | --- | --- | --- |
|  | *PaO_2_/FiO_2_ ratio model* | *ROX index model* | *ROX index routine model* | *ROX index pre-hospital model* | *PaO_2_/FiO_2_ ratio model* | *ROX index model* | *ROX index routine model* | *ROX index pre-hospital model* |  |  |  |  |  |  |  |  |  |  |
|  | **Safe discharge** | | | | | | | |  |  |  |  |  |  |  |  |  |  |
| DT | 0.937 | 0.919 | 0.500 | 0.500 | 0.858 | 0.778 | 0.742 | 0.746 |  |  |  |  |  |  |  |  |  |  |
| RF | 0.938 | **0.964** | **0.901** | 0.849 | **0.894** | 0.879 | **0.819** | 0.751 |  |  |  |  |  |  |  |  |  |  |
| GBM | **0.943** | 0.958 | 0.787 | **0.861** | 0.882 | **0.898** | 0.795 | **0.758** |  |  |  |  |  |  |  |  |  |  |
|  | **Disease severity** | | | | | | | |  |  | |  | **Disease severity** | | **Disease severity** | | | |
| DT | 0.792 | 0.824 | 0.810 | 0.799 | 0.766 | 0.709 | 0.726 | 0.623 |  |  |  |  |  |  |  |  |  |  |
| RF | 0.886 | 0.906 | **0.876** | **0.867** | **0.832** | **0.806** | **0.786** | 0.722 |  |  |  |  |  |  |  |  |  |  |
| GBM | **0.893** | **0.917** | 0.872 | 0.846 | 0.827 | 0.800 | 0.771 | **0.727** |  |  |  |  |  |  |  |  |  |  |
|  | **Mortality** | | | | | | | |  | | **Mortality** | | |  | | **Mortality** | **Mortality** | **Mortality** |
| DT | 0.758 | 0.708 | 0.778 | 0.698 | 0.689 | 0.735 | 0.730 | 0.725 |  |  |  |  |  |  |  |  |  |  |
| RF | **0.866** | **0.906** | **0.902** | **0.858** | 0.842 | 0.860 | **0.884** | 0.824 |  |  |  |  |  |  |  |  |  |  |
| GBM | 0.857 | 0.888 | 0.875 | **0.858** | **0.844** | **0.844** | 0.865 | **0.864** |  |  |  |  |  |  |  |  |  |  |

**Table 4.** ROC curves of the best models (in terms of AUC) including complete cases and missing data imputation process.


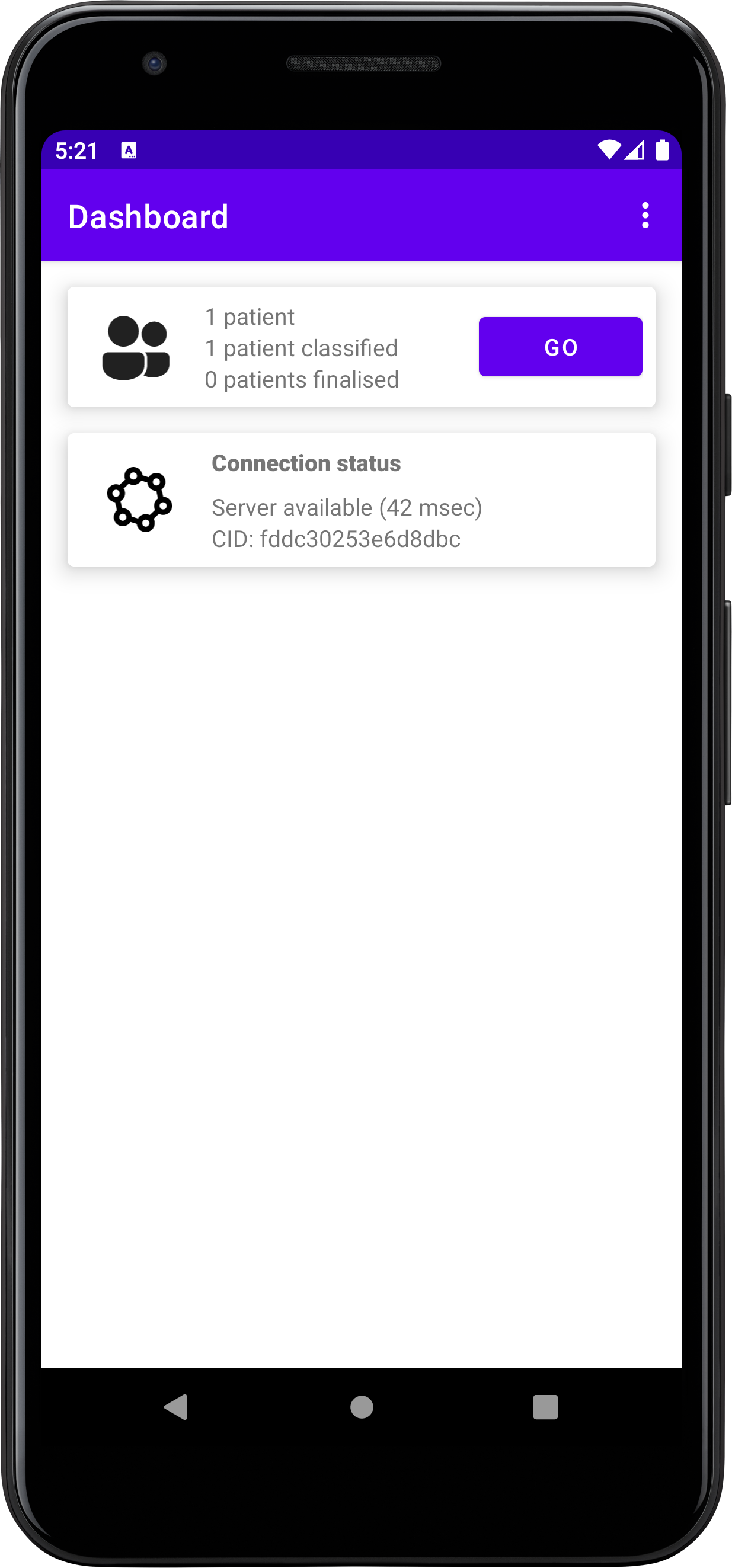

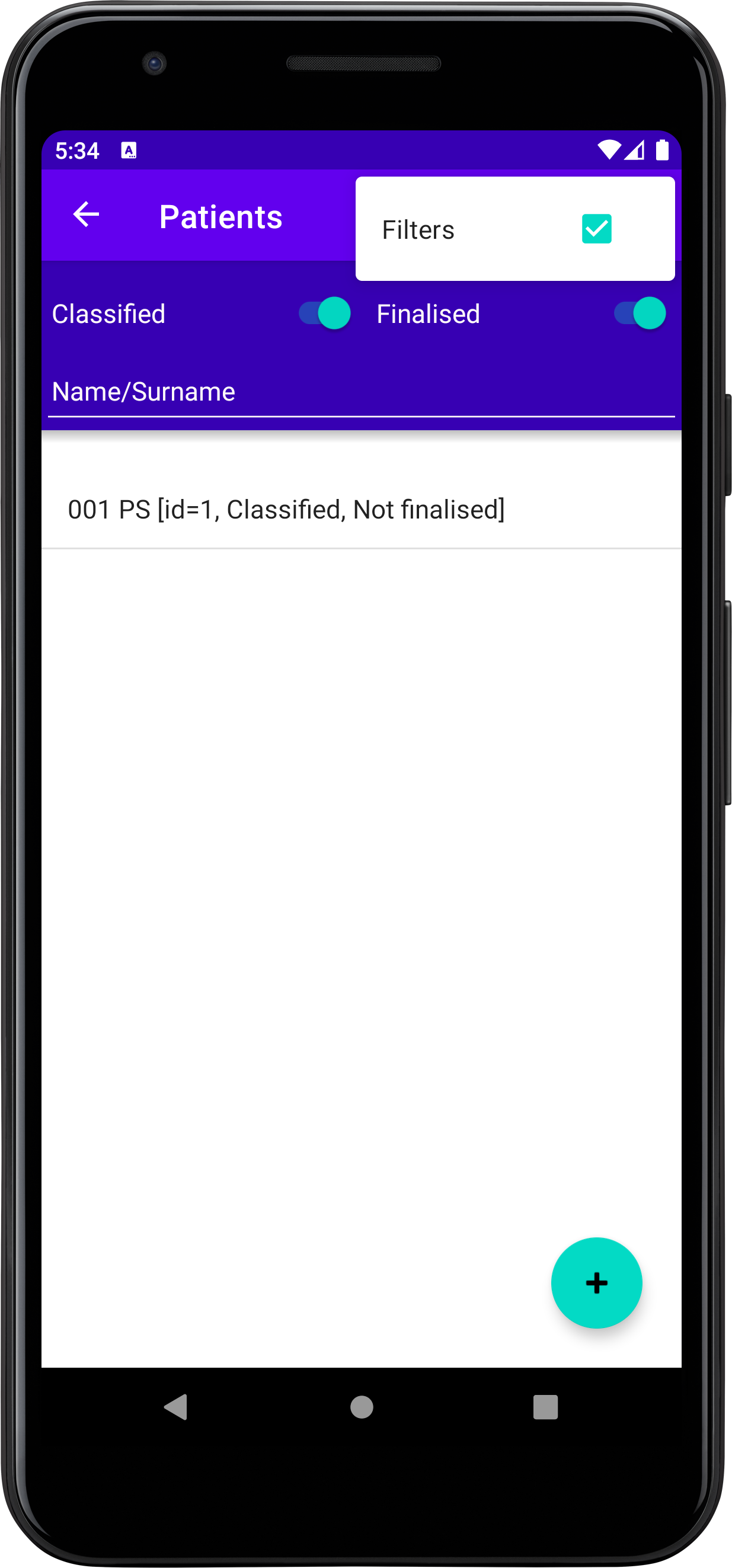

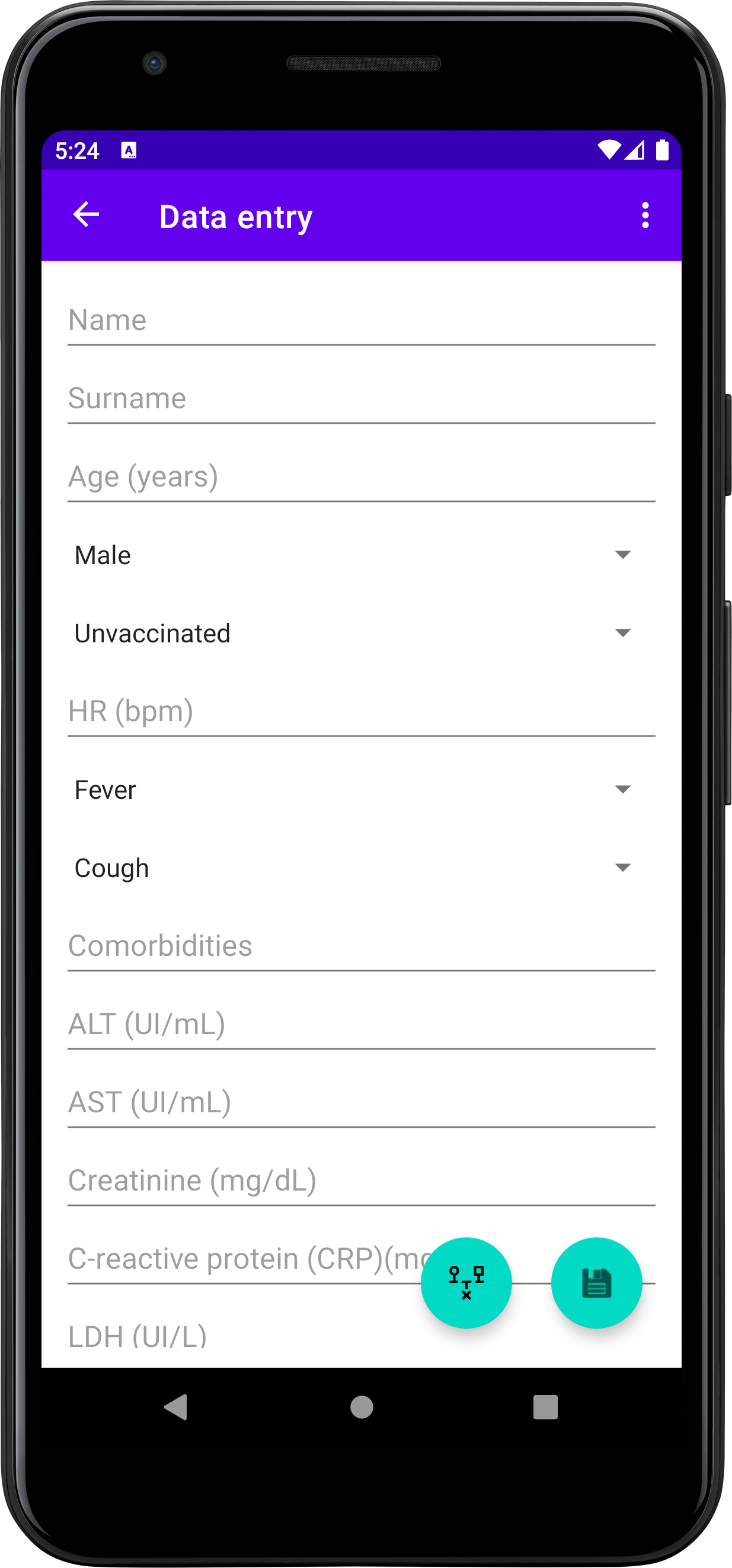

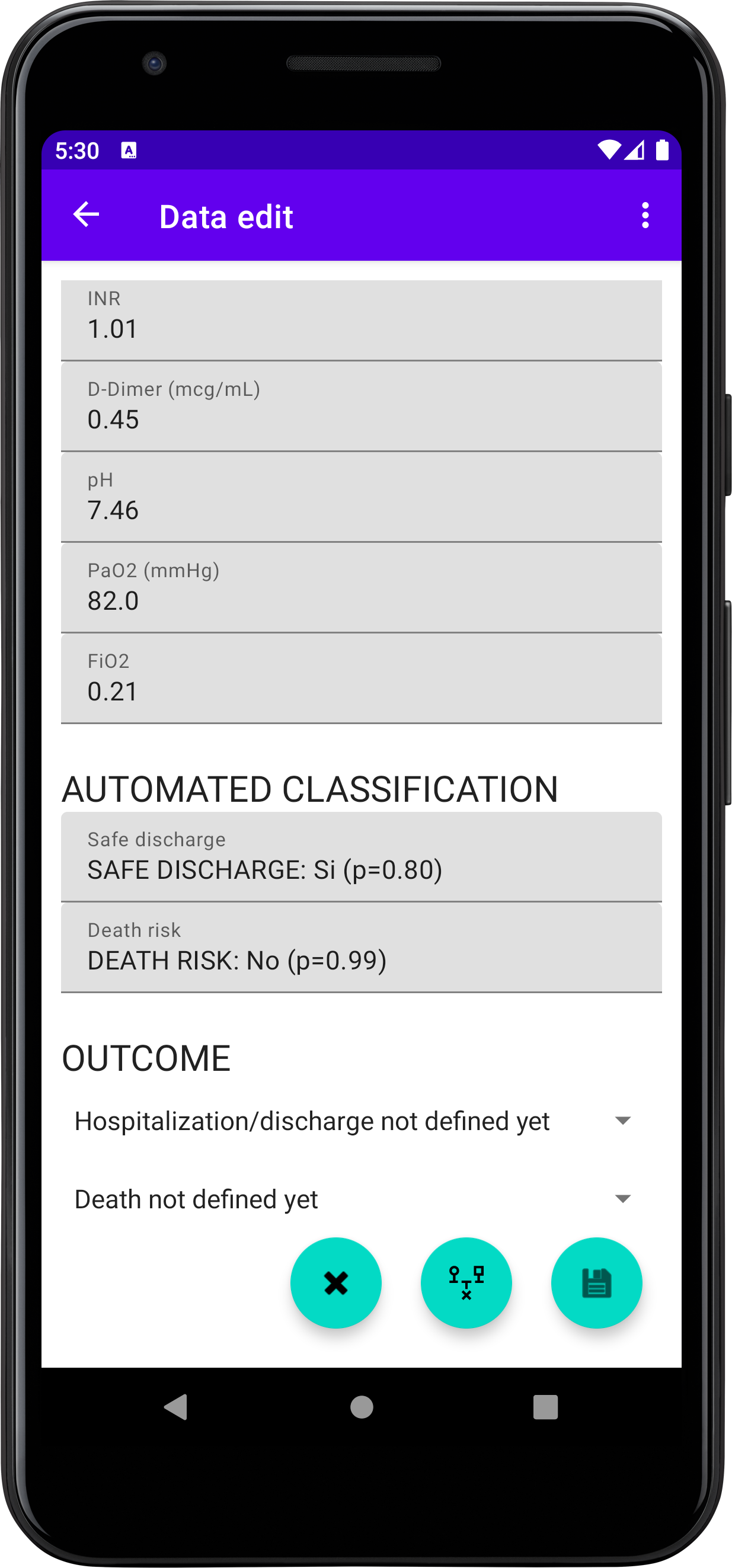


**(a) (b) (c) (d)**

**Figure 1. C19DSS activities. (a)** Dashboard, **(b)** List of patients, **(c)** New patient, **(d)** Edit patient data
